# Workload reduction of digital breast tomosynthesis screening using artificial intelligence and synthetic mammography: a simulation study

**DOI:** 10.1117/1.JMI.12.S2.S22005

**Published:** 2025-04-30

**Authors:** Victor Dahlblom, Magnus Dustler, Sophia Zackrisson, Anders Tingberg

**Affiliations:** a Lund University, Diagnostic Radiology, Department of Translational Medicine, Malmö, Sweden; b Skåne University Hospital, Department of Medical Imaging and Physiology, Malmö, Sweden; c Lund University, Medical Radiation Physics, Department of Translational Medicine, Malmö, Sweden; d Skåne University Hospital, Radiation Physics, Malmö, Sweden

**Keywords:** artificial intelligence, digital breast tomosynthesis, synthetic mammography, breast cancer screening

## Abstract

**Purpose:**

To achieve the high sensitivity of digital breast tomosynthesis (DBT), a time-consuming reading is necessary. However, synthetic mammography (SM) images, equivalent to digital mammography (DM), can be generated from DBT images. SM is faster to read and might be sufficient in many cases. We investigate using artificial intelligence (AI) to stratify examinations into reading of either SM or DBT to minimize workload and maximize accuracy.

**Approach:**

This is a retrospective study based on double-read paired DM and one-view DBT from the Malmö Breast Tomosynthesis Screening Trial. DBT examinations were analyzed with the cancer detection AI system ScreenPoint Transpara 1.7. For low-risk examinations, SM reading was simulated by assuming equality with DM reading. For high-risk examinations, the DBT reading results were used. Different combinations of single and double reading were studied.

**Results:**

By double-reading the DBT of 30% (4452/14,772) of the cases with the highest risk, and single-reading SM for the rest, 122 cancers would be detected with the same reading workload as DM double reading. That is 28% (27/95) more cancers would be detected than with DM double reading, and in total, 96% (122/127) of the cancers detectable with full DBT double reading would be found.

**Conclusions:**

In a DBT-based screening program, AI could be used to select high-risk cases where the reading of DBT is valuable, whereas SM is sufficient for low-risk cases. Substantially, more cancers could be detected compared with DM only, with only a limited increase in reading workload. Prospective studies are necessary.

## 1 Introduction

Digital mammography (DM) has long been the standard method for breast cancer screening. Screening with digital breast tomosynthesis (DBT) has been shown to have a higher sensitivity for cancer in several studies.^1^^–^^6^ As DBT results in many more images to be interpreted than DM, the reading time per examination is longer—in previous studies often 70% to 100% longer than for DM, but it differs depending on factors such as reading protocol and the DBT reading experience of the readers.^7^^–^^10^ In screening programs operating with high recall levels, e.g., in the United States, the use of DBT has also been shown to decrease the recall rates.^11^^,^^12^ As unnecessary recalls can cause anxiety,^13^ this is advantageous for the screened women but might also counteract the increased reading time for DBT as each recall requires substantial extra time and resources on top of the screen reading.^14^ In studies from Europe, where the screening programs generally employ double reading and operate with lower recall levels, the use of DBT has had more varied effects on the recall rate, and both slight decreases and increases have been observed.^3^^,^^12^^,^^15^ DBT is increasingly used in screening in the United States,^16^^,^^17^ but European screening programs have been more hesitant to introduce DBT in screening, often motivated by the incapability to accommodate the increased reading workload due to the shortage of breast radiologists.^18^^,^^19^ Single reading of DBT has been shown to detect more cancers than double reading of DM and only slightly fewer cancers than double reading of DBT.^4^^,^^20^

In recent years, several artificial intelligence (AI) systems have been introduced aimed at supporting breast cancer screening, initially mainly for decision support in the reading situation but increasingly also for managing the workflow. Using AI to exclude normal cases from human reading, both for DM and DBT screening, has been suggested.^21^^–^^27^ However, studies on opinions about AI among women attending screening have found that human involvement in all decisions is expected if AI is used.^28^^–^^31^ Furthermore, there might be legal issues regarding the responsibility for decisions taken by the AI system alone.^32^

In a previous study, we have shown that in DM-based screening, AI can be used to automatically add DBT in selected high-risk cases, thereby detecting a majority of the breast cancers detected by DBT only.^33^ However, not all DBT-detected cancers would be detected, and adding a DBT examination for some women would probably cause logistical challenges at screening centers; thus, a DBT-based screening for all women would probably be preferable.

Synthetic mammography (SM) is a method where a two-dimensional image resembling a DM examination is generated based on a DBT examination. It can be useful, for example, to compare with previous examinations performed with DM but also to get a quick overview of the examination. The properties of the DBT vary among vendors, such as sweep angle, which affects the complexity of generating an SM image based on the DBT data.^34^ SM has been shown to be a sufficient or even superior alternative to DM in several retrospective studies,^35^^–^^43^ although in some cases non-significantly inferior sensitivity values have been reported.^39^^,^^44^ Most of the available studies of SM focus on using it in combination with DBT reading,^35^^,^^36^ but a few studies have compared reading of SM and DM alone.^37^^–^^41^ Furthermore, most studies have used Hologic narrow-angle systems. One study using a FUJIFILM system (in narrow-angle mode) showed higher cancer detection on an AI-generated SM examination, where suspicious findings are enhanced in the SM image, than in the corresponding DM examination.^45^ In one small study, SM based on wide-angle DBT systems from Siemens has been compared with DM without finding any substantial differences in cancer detection and no significant differences in the area under the receiver operating characteristics curve.^43^ Furthermore, among 10 image quality criteria, no significant differences were found between SM and DM except for distinguishing the gland from adipose tissue and the sharpness of ligament duct vessels. Another study also using a Siemens wide-angle system showed the superiority of combined DBT and SM compared with DM alone.^42^

The hypothesis of this study is that if all women are screened with DBT, an AI system can identify high-risk cases, where the full DBT examination should be read, and low-risk cases where reading only the SM examination is sufficient. We have investigated this by analyzing screening DBT examinations with an AI system, and—based on the AI score—we have used either the original DBT reading results or the DM reading results. The DM reading results were used as a surrogate for SM reading results.

## 2 Methods

### 2.1 Study Design and Study Population

**Fig. 1:**
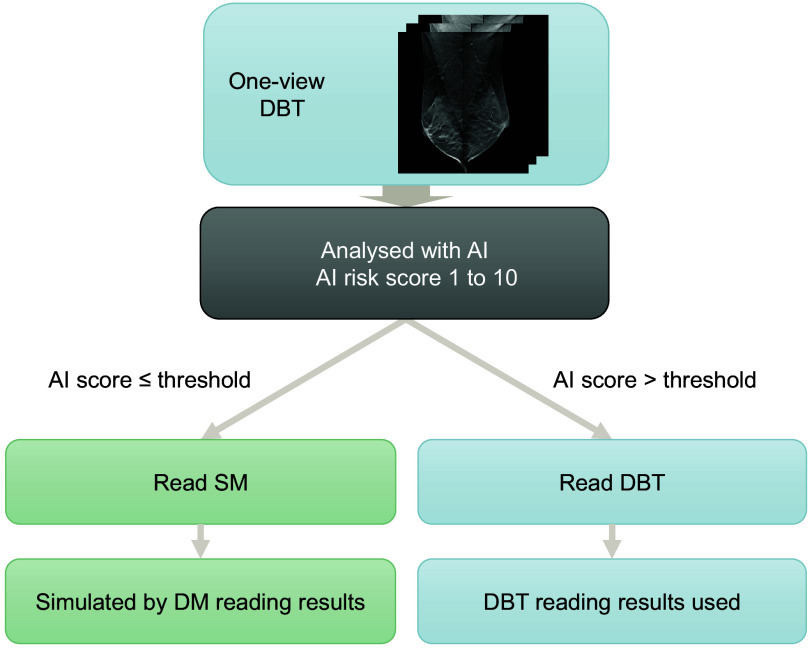
Illustration of proposed workflow.

This is a simulation study exploring the potential of using a cancer detection AI system to triage DBT screening examinations to reading of either the SM examination in low-risk cases or the full DBT examination in high-risk cases, as illustrated in Fig. 1. The study is based on the Malmö Breast Tomosynthesis Screening Trial (MBTST), which included 14,848 women who were examined with both two-view DM [craniocaudal and mediolateral oblique (MLO)] and one-view wide-angle DBT (MLO) at the same appointment.^3^ All examinations were performed with a Siemens Mammomat Inspiration breast imaging machine (Siemens Healthcare GmbH, Erlangen, Germany). The DM and DBT examinations were separately double-read, and consensus meetings were held before recalls. Reading data from the first reader on DM and DBT were extracted to simulate a single-reading situation. For a single reader, the cases were considered to be recalled if they were marked for recall or discussion by the first reader and recalled after the consensus meeting. The reading results from the DM reading were used to simulate SM reading results, as no SM images or readings were performed in the MBTST. No AI system was used in the reading situation. A few examinations in the MBTST had to be excluded from this study due to missing or corrupted data, giving a total number of 14,772 included women. This study is covered by the study approval for the MBTST by the Local Ethics Committee at Lund University (Official Records No. 2009/770).

### 2.2 AI System

The DBT examinations were analyzed with the cancer detection system ScreenPoint Transpara 1.7 (ScreenPoint Medical, Nijmegen, the Netherlands).^22^^,^^26^^,^^46^^–^^49^ The system gives each examination a score (called the AI score in this paper) between 0 and 10, where 10 means the highest risk of cancer. In the user interface of the AI system, this is rounded up to an integer between 1 and 10, but for this study, the raw continuous decimal score was used. No DM or SM was used in the analyses by the AI system.

### 2.3 Analyses

The workload, number of recalls, and detected cancers were quantified for each of the four combinations of single-reading and double-reading SM and DBT, with AI score thresholds varied continuously between 0 and 10. Two specific thresholds were selected and analyzed more thoroughly. The first threshold was where an unchanged reading workload was achieved compared with DM double reading. The second threshold was where the reading workload was reduced slightly compared with DM double reading while the cancer detection was more clearly increased.

As the actual reading times were not registered in the MBTST, data on reading time for SM and DBT from previous studies were used. The reading times varied among studies, with a span of 25 to 59 s for DM (assumed to be equivalent to SM), 44 to 71 s for DBT alone, and 91 to 125 s for DBT combined with DM.^7^^–^^10^ However, the studies reported reading times for DBT that were approximately twice as long as for DM. Thus, the workload was approximated by assuming a double-reading time with DBT compared with SM. The time for single reading an SM examination was defined as one reading time equivalent, and thus, double reading an SM examination or single reading a DBT examination equaled two reading time equivalents, whereas double-reading a DBT examination equaled four reading time equivalents.

The results were analyzed with descriptive statistics. All analyses were performed in MATLAB 2023b (The MathWorks, Natick, Massachusetts, United States).

## 3 Results

**Fig. 2:**
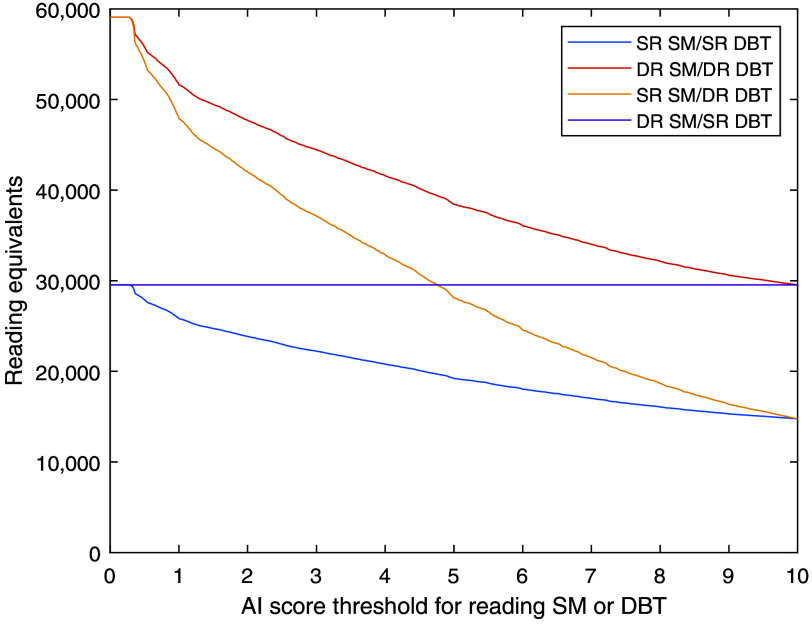
Workload at different thresholds for reading SM or DBT in relation to DBT double reading. SR, single reading; DR, double reading. The workload for reading a DBT examination is defined as twice the workload for reading an SM examination. Thus, the reading time in the DR SM/SR DBT approach would not be affected by the reading threshold and the graph equals the current uniform DM double reading.

The workload, measured in reading equivalents, as simulated based on the MBTST data for the different combinations of single-reading or double-reading SM and DBT depending on the AI score threshold, is shown in Fig. 2. The SR SM/DR DBT approach, in which the radiologists focus most of the reading time on the examinations that the AI system identified as showing the highest risk, crosses the DR SM/SR DBT graph at a threshold of ∼5. Thus, this threshold would give a largely unchanged total workload compared with double-reading DM.

**Fig. 3:**
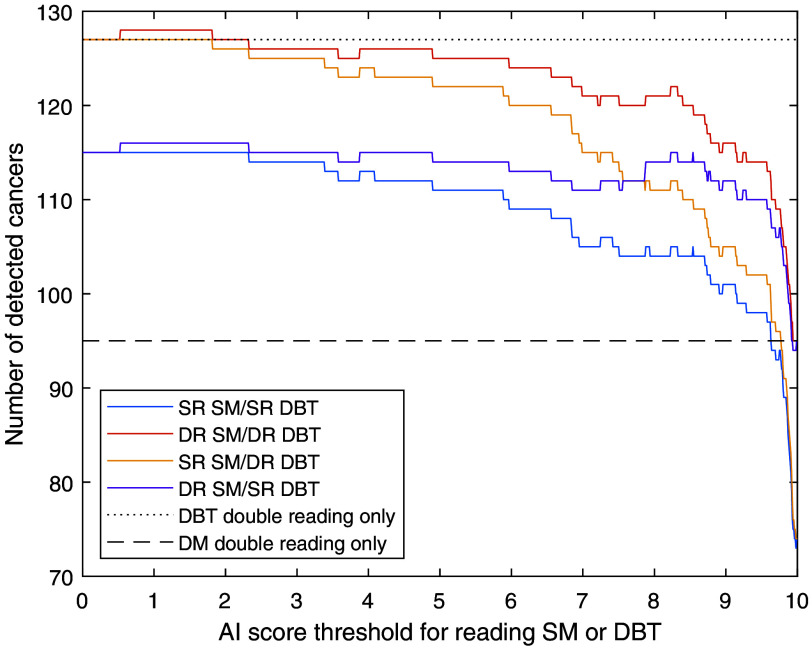
Number of detected cancers at different thresholds for reading SM or DBT in relation to DBT double reading. SR, single reading; DR, double reading.

The corresponding variation in cancer detection is shown in Fig. 3. As more cancers were detected on DBT, workflows where many DBT examinations are read are usually favorable. The most labor-intensive approach with a double reading of both SM and DBT has the highest cancer detection, but with an AI threshold at 5, the SR SM/DR DBT approach has only a slightly lower cancer detection (8.26 versus 8.46 detected cancers per 1000 screened women).

**Fig. 4:**
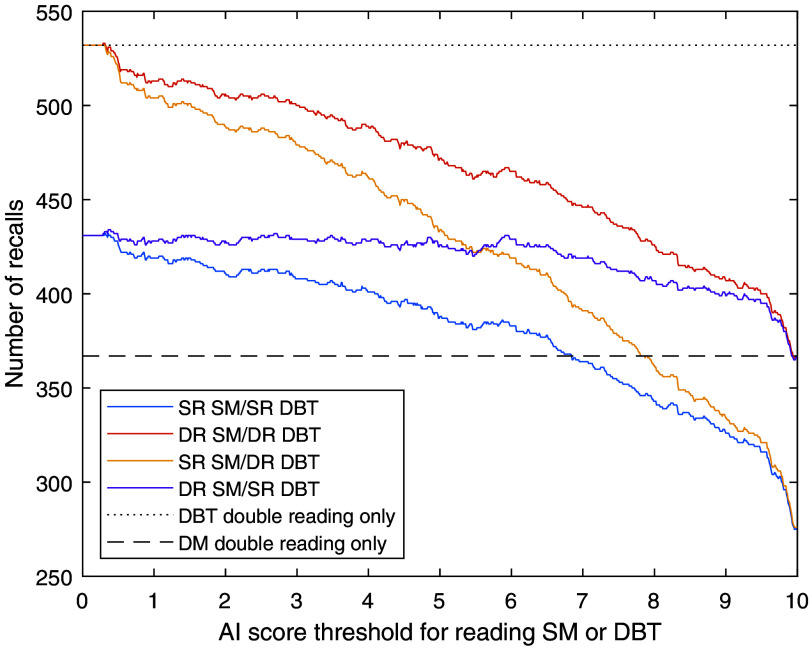
Number of recalls at different thresholds for reading SM or DBT in relation to DBT double reading. SR, single reading; DR, double reading.

In Fig. 4, the number of recalls for the different reading approaches and AI score thresholds is shown. Due to the underlying higher recall rate for DBT, most of the reading approaches have a higher recall rate than DM double reading. However, at a threshold of 7 and above, the SR SM/SR DBT approach gives a decreased recall rate. At the same threshold, the SR SM/DR DBT approach has only a slight increase of 6.5%, where 83% of additional recalls correspond to additional detected cancers.

**Fig. 5:**
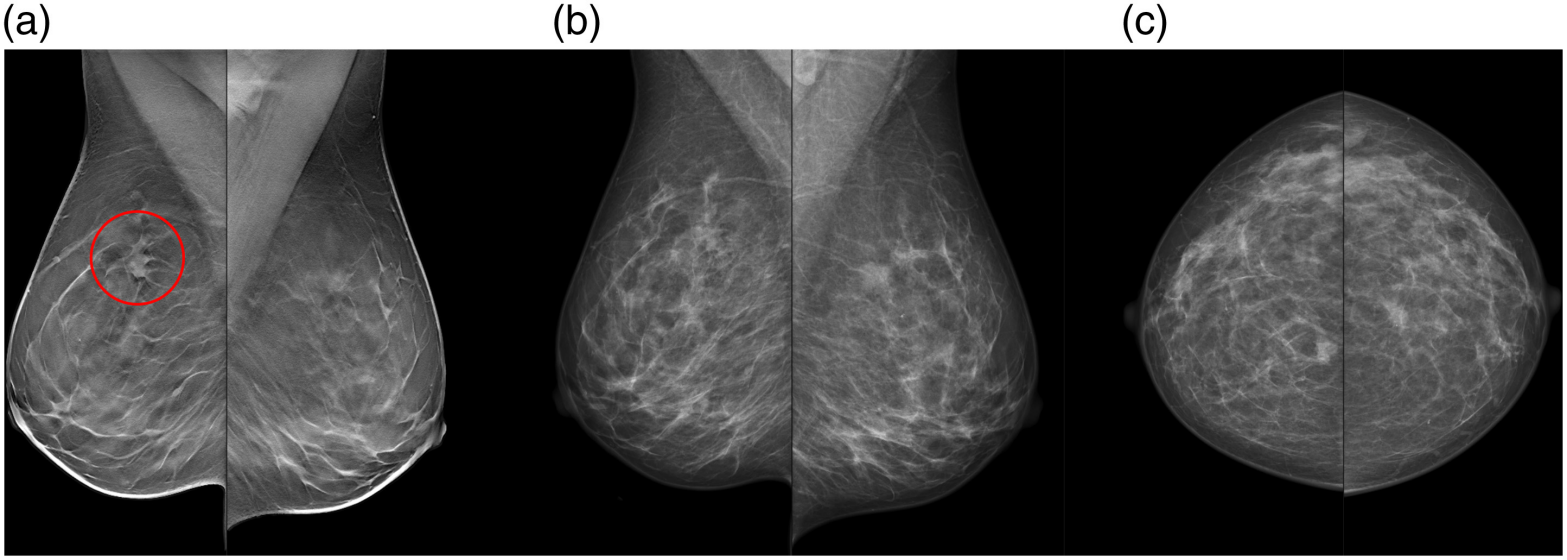
Cancer (red circle) non-detected with DM double reading but detected with DBT screening and that would also be detected if the DBTs were only read in high-risk cases identified by the AI system. The AI score was 9.86, and the cancer was a 12-mm invasive ductal carcinoma/invasive carcinoma of no special type. (a) Mediolateral oblique DBT. (b) Mediolateral oblique DM. (c) Craniocaudal DM.

More details on the number of read examinations, workload, recalls, false-positive recalls, detected cancers, and non-detected cancers are presented for the thresholds of 5 and 7 in Tables 1 and 2, respectively. In these tables, the numbers are also presented separately for the SM and DBT parts of each of the workflows, i.e., below and above the threshold. In total, 135 cancers were detected through double reading of both DM and DBT combined, 95 through double reading of DM, and 127 through double reading of DBT. With a threshold of 5 means that, by double reading the DBT examination of 30% (4452/14,772) of the cases with the highest risk and single reading the SM examination for the rest, 122 cancers would be detected with the same reading workload as DM double reading. That is, 28% (27/95) more cancers than with DM double reading and in total 96% (122/127) of the cancers detectable with full DBT double reading would be found. If using a less conservative assumption of a 70% longer reading time for DBT than DM/SM, this approach would reduce the reading workload by 14% compared with DM double reading. Figure 5 shows an example of a cancer that was not detected by DM double reading but would be detected if DBTs were read in cases with high AI scores.

**Table 1:** Read DBT if the AI score is 5 or higher (DBT examinations are read for 30% of the women)—workload at the same level as DM double reading with single-reading SM + double-reading DBT.

		Read examinations	Workload	Recalls	False-positive recalls	Detected	Non-detected^a^
Single-reading SM + single-reading DBT	SM	10,320	10,320	107	101	6	7
	DBT	4452	8904	281	176	105	17
	Total	14,772	19,224	388	277	111	24
Single-reading SM + double-reading DBT	SM	10,320	10,320	107	101	6	7
	DBT	4452	17,808	327	211	116	6
	Total	14,772	28,128	434	312	122	13
Double-reading SM + single-reading DBT	SM	10,320	20,640	145	136	9	4
	DBT	4452	8904	281	176	105	17
	Total	14,772	29,544	426	312	114	21
Double-reading SM + double-reading DBT	SM	10,320	20,640	145	136	9	4
	DBT	4452	17,808	327	211	116	6
	Total	14,772	38,448	472	347	125	10

a Compared with the 135 cancers detected with combined DM and DBT double reading.

**Table 2:** Read DBT if the AI score is 7 or higher (DBT examinations are read for 22% of the women).

		Read examinations	Workload	Recalls	False-positive recalls	Detected	Non-detected^a^
Single-reading SM + single-reading DBT	SM	11,505	11,505	131	123	8	9
	DBT	3267	6534	252	151	101	17
	Total	14,772	18,039	383	274	109	26
Single-reading SM + double-reading DBT	SM	11,505	11,505	131	123	8	9
	DBT	3267	13,068	288	176	112	6
	Total	14,772	24,573	419	299	120	15
Double-reading SM + single-reading DBT	SM	11,505	23,010	177	165	12	5
	DBT	3267	6534	252	151	101	17
	Total	14,772	29,544	429	316	113	22
Double-reading SM + double-reading DBT	SM	11,505	23,010	177	165	12	5
	DBT	3267	13,068	288	176	112	6
	Total	14,772	36,078	465	341	124	11

a Compared with the 135 cancers detected with combined DM and DBT double reading.

## 4 Discussion

We have shown that AI has the potential to mitigate the increased reading workload caused by the transition from DM to DBT in screening by separating high-risk examinations, where the reading of the DBT examination is necessary, from low-risk examinations, where reading of the SM examination is sufficient. By focusing the reading effort on the most complex cases, most of the advantages of DBT screening can be achieved, whereas keeping a radiologist reading of the SM examination in low-risk cases would still fulfill the demand for human reading from women taking part in screening and would also meet potential legal requirements.

This study was based on a previously collected dataset with paired DM and DBT examinations. Thus, no actual reading of SM examinations was performed; instead, the DM reading results were used as surrogates. This assumes that SM is equivalent to DM, which is only shown to a limited extent by previous studies and only in retrospective data.^35^^,^^37^^–^^43^ If SM would prove not to actually reach the cancer detection level of DM, the number of detected cancers might be slightly lower than found in this simulation study. However, unless a very high threshold for reading DBT is selected, relatively few cancers are detected among the SM-read cases, and thus, the effect should be limited. If SM on the other hand would prove to be better than DM, it might be possible to use a higher threshold and get a larger reduction in workload.

In the MBTST, a few cancer cases were detected on DM but not on DBT, potentially due to different positioning, better possibilities for comparison with previous examinations with DM, and random reader variation. This means that a few cancers cannot be detected by the DBT workflows (as simulated in this paper), and thus, a few cancers are not detected, regardless of workflow and threshold (Tables 1 and 2). This also explains some of the small local maxima in Fig. 3.

**Fig. 6:**
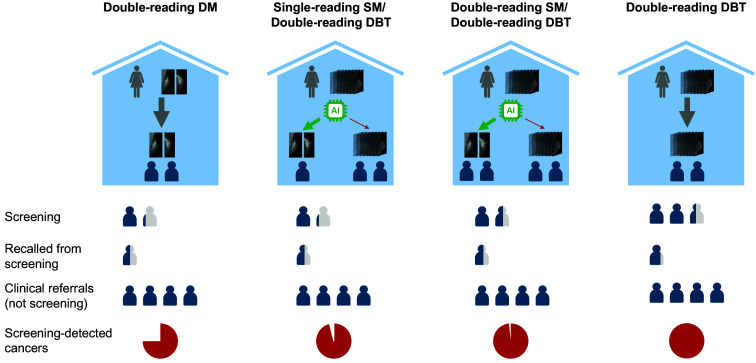
Example of effects on the workload at a breast radiology clinic for different workflows using DM, DBT, and SM. Two AI-enhanced workflows are included (where an AI score threshold of 5 is used for triaging between SM and DBT): single-reading SM in low-risk cases and double-reading DBT in high-risk cases (SR SM/DR DBT) and double-reading SM in low-risk cases and double-reading DBT in high-risk cases (DR SM/DR DBT). Double-reading DM (current routine) and double-reading DBT with no AI triage are included for comparison. The number of full-time-equivalent breast radiologists necessary for handling each of the following tasks is presented: reading of screening examinations, examination of women recalled from screening, and clinical referrals from other clinics due to symptoms, etc. The proportion of screening-detected cancers relative to double-reading DBT is presented as pie charts. The example is based on the following assumptions about the breast radiology clinic: 68,000 screening examinations/year. A reading time of 40 s for DM/SM and 80 s for DBT. Estimations of the number of breast radiologists were based on Swedish work hours (40 h a week) and a capacity of 20 recalled or clinically referred women per full breast radiologist day. About 10,500 women per year are referred (not screening) to the breast radiology clinic due to symptoms etc., and this flow is assumed to be unaffected by the screening program.

The optimal choice among the studied combinations of double and single reading and threshold might vary depending on the current characteristics of the screening program, local regulations, and the workload that can be handled by available readers. Double reading of both DBT (for high-risk cases) and SM (for low-risk cases) gives the highest workload among the studied workflows but detects the most cancers and still saves substantial work compared with double-reading all DBT examinations. It would also fulfill any potential legal requirements of using double reading that might apply to some screening programs. The largest reduction in workload would be reached using single reading for both SM and DBT and might be the most relevant approach in screening programs where single reading is currently the standard. If possible with respect to resources, the workflow with SM single reading and DBT double reading is probably the best among the studied workflows with a good balance between many detected cancers and a limited workload. The combination of double-reading low-risk SM and single-reading high-risk DBT gives an unchanged reading workload but is not clinically relevant as much work is required for double-reading low-risk cases and it would be better to instead single-read DBT for all examinations without any AI sorting. Regarding the threshold, a threshold of 5 in the single-reading SM/double-reading DBT workflow, thereby reading DBT on 30% of the examinations, would be workload-neutral compared with the current widespread routine of double-reading DM. If some reduction of workload is needed, a threshold of 7 would further reduce the workload (17% less than DM double reading) with a limited reduction in cancer detection. For thresholds above 7.5, the proportion of examinations where only SM is read is so high that the SM-reading part dominates the results. This means that there is a threshold range where the DR SM/SR DBT workflow detects more cancers than the SR DM/DR DBT workflow (Fig. 3), but this approach is still inferior as the workload for DR SM/SR DBT is also higher for these scores (Fig. 2). Even higher score thresholds are not very useful, regardless of approach, as very little would be gained from the DBT examinations. An illustration of the effects on an example breast clinic of using some of the studied workflows is presented in Fig. 6.

Due to the large workload caused by each recalled woman, the recall rate is of paramount importance for the total workload of the screening program. In this study, most of the studied scenarios increased the recall rate to some extent. This was due to the higher recall rate for DBT than for DM in the underlying MBTST.^3^ At least to some extent this seemed to be related to reader experience with DBT, and the increase would probably diminish after the initial phase. However, as seen in the example in Fig. 6, the effect of a slight increase in recall rate on the overall workload in a breast radiology clinic might be relatively limited, as the number of women who are admitted due to symptoms is much larger than the number of women recalled from screening.

There are several other potential ways of using AI to handle the longer reading time with DBT. It has been proposed to use AI to identify high-risk cases that should be double-read, whereas the low-risk cases can be either single-read or completely discarded for human reading.^22^^,^^27^ In this study, no AI results were available to the readers, as the analyses by the AI system were performed retrospectively. In a real screening program, it would be possible to let the readers use the AI systems’ results in the reading situation. Two recently published prospective DM screening studies investigated the difference between traditional double reading without AI and single reading with AI, one using AI as an independent reader by blinding the radiologist to the AI results, and one using the AI system as a support tool to the radiologist. The study where the radiologist and AI system were allowed to interact showed slightly better results, suggesting that this approach might be superior.^50^^,^^51^ A similar relation for DBT is likely, but this has not yet been studied for DBT. Thus, if implementing AI to triage between reading DBT and SM in a real screening program, it might be preferable to have a single reader using an AI system as a support tool, rather than using AI as a separate reader. Another alternative could be to continue with primarily a DM-based screening but use AI to identify women for whom adding DBT would have a high gain.^33^ However, this would probably cause logistical challenges at screening centers and would still not achieve the full effect of DBT screening.

There are several limitations to this study, most of which are inherent in the retrospective design. The AI results were not available during the reading, which probably affected the results. Updated versions of the AI system have been released, and it is possible that using these might give different results. The score level of the threshold corresponding to a specific proportion of examinations to be DBT read might vary with the version and properties of the analyzed examinations. Furthermore, the usage of one-view DBT in this study caused a general shift to lower scores.

The SM readings were surrogated by the original DM readings. Reading of the SM examination would be preferable to also assess the suitability of the SM images as a basis for reading. However, collecting such data in a retrospective setting would come with a risk of getting results that are non-representative of clinical work due to the laboratory effect.^52^ This study used one-view DBT and two-view DM, which would be unrealistic in a clinical context as it would be necessary to have two-view DBT to be able to generate two-view SM. As the DM and DBT examinations in the study were taken with slightly different positioning, in some cases, the difference between the DM examination and the hypothetical SM examination might be larger than the difference attributable specifically to the reconstruction of SM from DBT.

The actual reading time was not measured in the MBTST, and all calculations of reading times used assumptions based on data from other studies. This did not take into account the difference in reading time depending on the complexity of the examinations. Furthermore, the data from many of the previous studies were based on data from trials where the reading protocols might be more complex than in common screening reading practice. The reported reading times have a large variation, and the assumption of double reading time for DBT compared with DM might be overly conservative.

## 5 Conclusions

In this simulation study based on previously collected data, we have shown that an AI system has the potential to triage reading of breast cancer screening examinations between SM in low-risk cases and DBT in high-risk cases. This would give a 28% higher cancer detection without an increase in workload in the clinic compared with double-reading DM. In this exploratory study, the SM reading was surrogated with DM reading results. Further studies are needed, both to validate that using SM provides sufficient performance and to test the proposed workflows prospectively.

## Data Availability

This study is based on breast cancer screening data that are not publicly available due to privacy and ethical restrictions.
